# Supplementary material for: Single-cell replication profiling to measure stochastic variation in mammalian replication timing
Source: Nat Commun. 2018 Jan 30;9:427. doi: 10.1038/s41467-017-02800-w (PMC5789892; doi:10.1038/s41467-017-02800-w)
Supplement: Supplementary file 1 — Supplementary Information [file 41467_2017_2800_MOESM1_ESM.pdf]

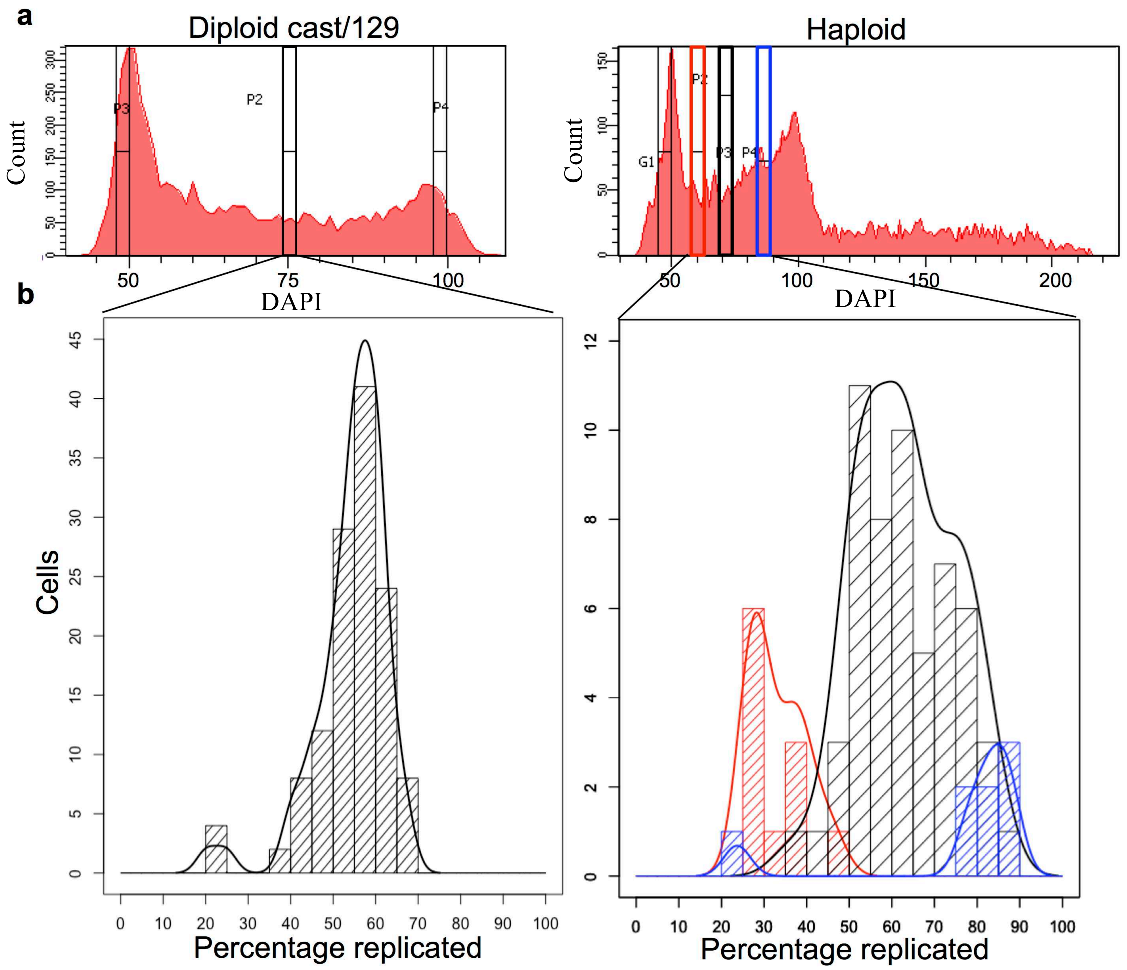

Supplementary Figure 1: Single-cell sequencing ranks cells accurately in S-phase. a) FACS (Fluorescence Activated Cell Sorting) profile based on DNA content and sorting gates used for diploid and haploid cells. Mid-S-phase cells were sorted for diploid cells. For haploid cells, 12 early S-phase cells (red gate), 12 late S-phase cells (blue gate) and 62 mid S-phase cells (black gate) were sorted. b) Ranking the cells in S-phase after binarization accurately reflects the position of cells in S-phase in both haploid and diploid cells. For reasons we don't understand, the FACS profile of haploid cells show a high proportion of late S-phase cells. Thus, while majority of the cells sorted using the mid-S-phase FACS gate (black gate) were middle replicating cells, there were also cells from later stages in S-phase. This is accurately reflected by the haploid S-phase rank histogram which shows a skew towards late S-phase. The skew was inconsequential and in fact provided a wider distribution of S-phase cells for subsequent analyses.

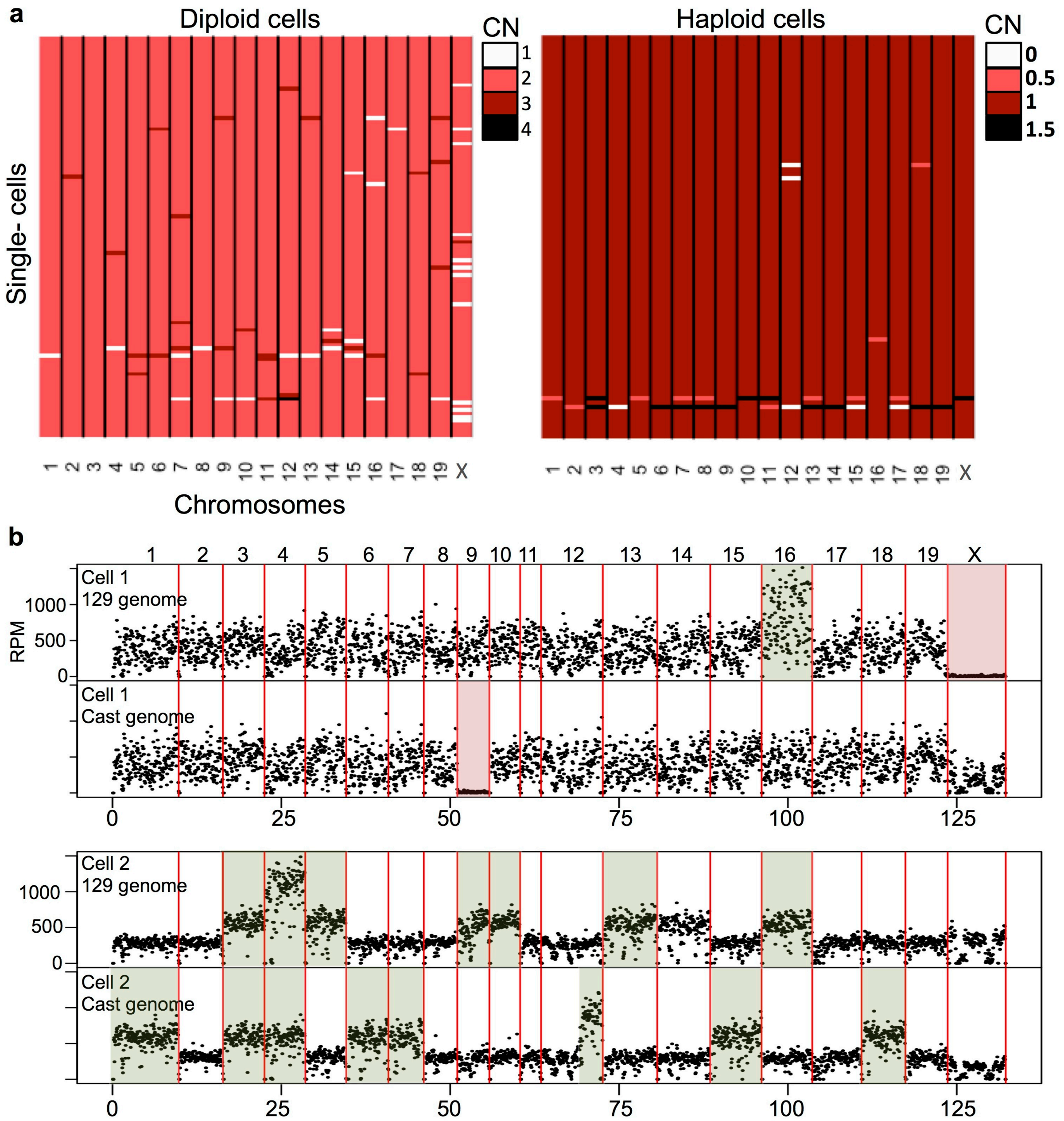

Supplementary Figure 2: Karyotype aberrations in diploid and haploid cells. a) Heat map of whole chromosome karyotype aberrations. Approximately 27 percentage of diploid cells show karyotype aberrations. The intermediate copy number observed in 4 haploid cells may be due to random diploidization. b) Example cells showing karyotype aberrations in both 129 and Castaneus genomes at 1 Mb resolution. Duplications are highlighted in green and deletions are highlighted in red.

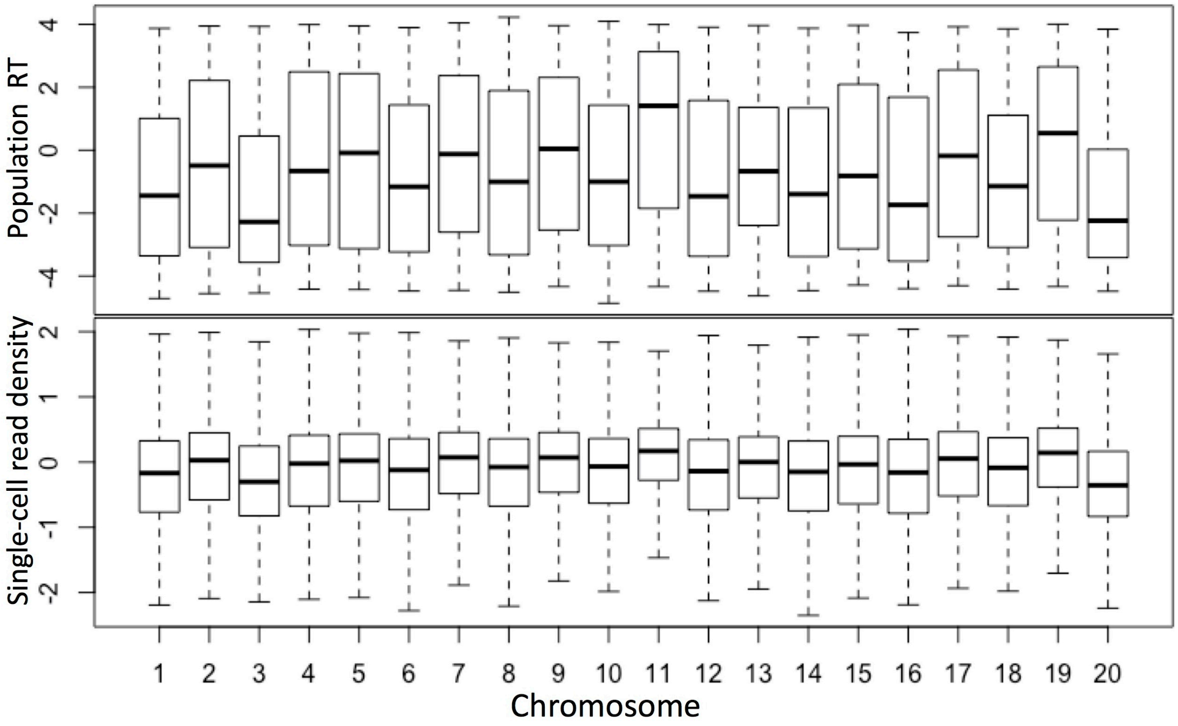

Supplementary Figure 3: Comparison between population RT and single-cell CNV signal. Top panel shows distribution of population-based replication timing values as measured by Repli-seq. Bottom panels shows smoothed normalized single-cell read density. Chromosomes skewed towards later replication have a lower distribution of read density and chromosomes skewed towards earlier replication have a higher distribution of read density.

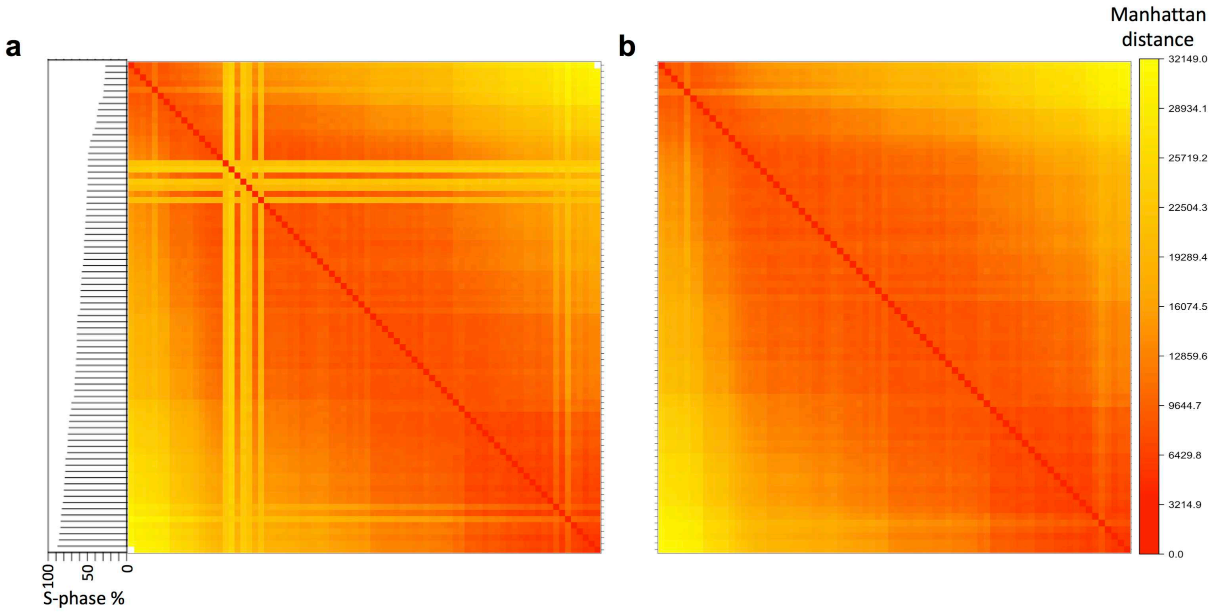

Supplementary Figure 4: Removing outlier cells. a) Heat map of genome-wide Manhattan distance using binarized data for every pair-wise combination of haploid cell ordered by their rank in S-phase. The ranking is shown as a barplot on the left. Outlier cells that do not correlate with any other cells appear as streaks on the heat map. b) Heat map after removing the outlier cells.

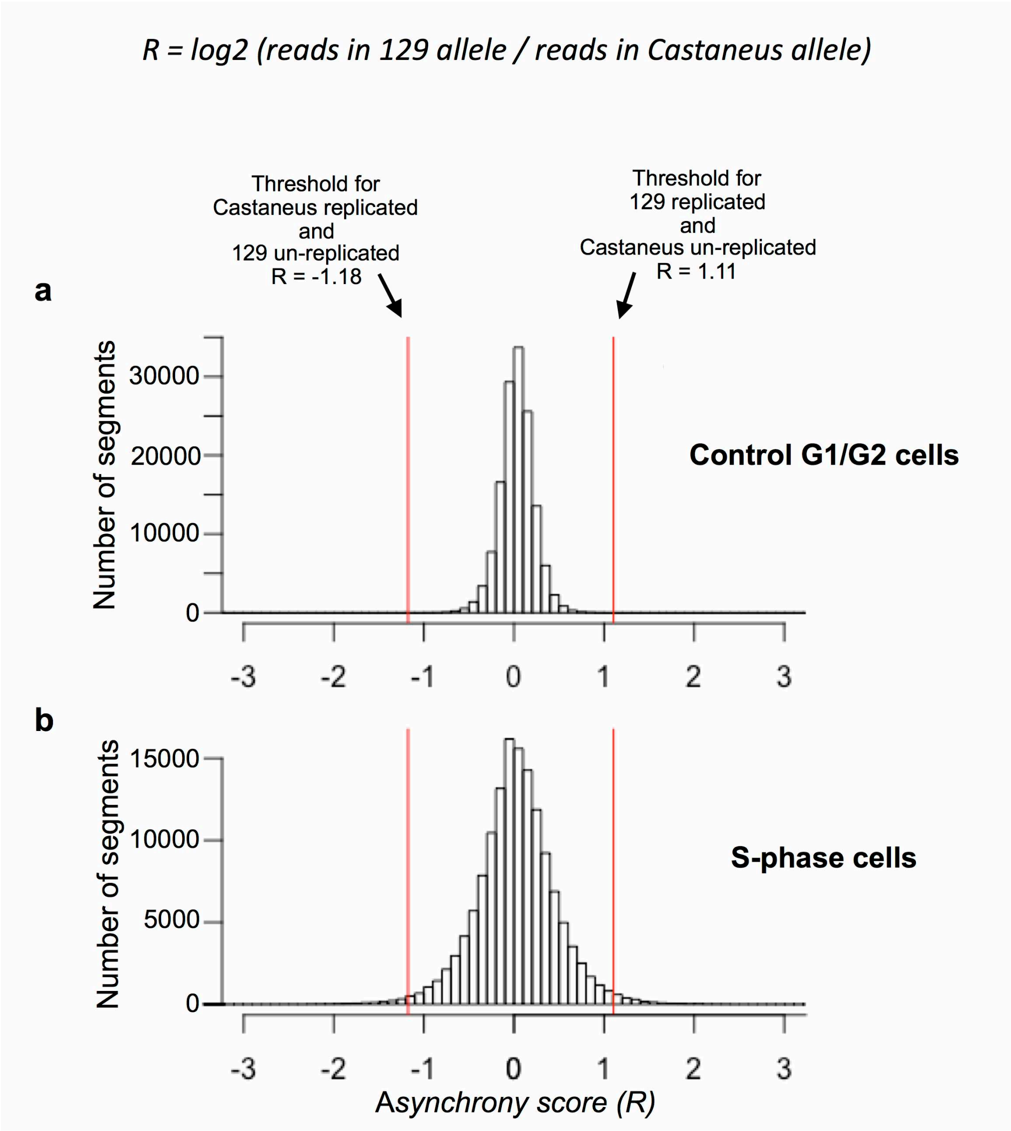

Supplementary Figure 5: Calculating the threshold asynchrony score to define segments in diploid cells that show homologue asynchrony a) Histogram of asynchrony score (R) calculated using the control G1/G2 cells. b) Histogram of asynchrony score in S-phase cells. The thresholds (red lines) were set to be the maximum and minimum asynchrony score in the control G1/G2 cells.

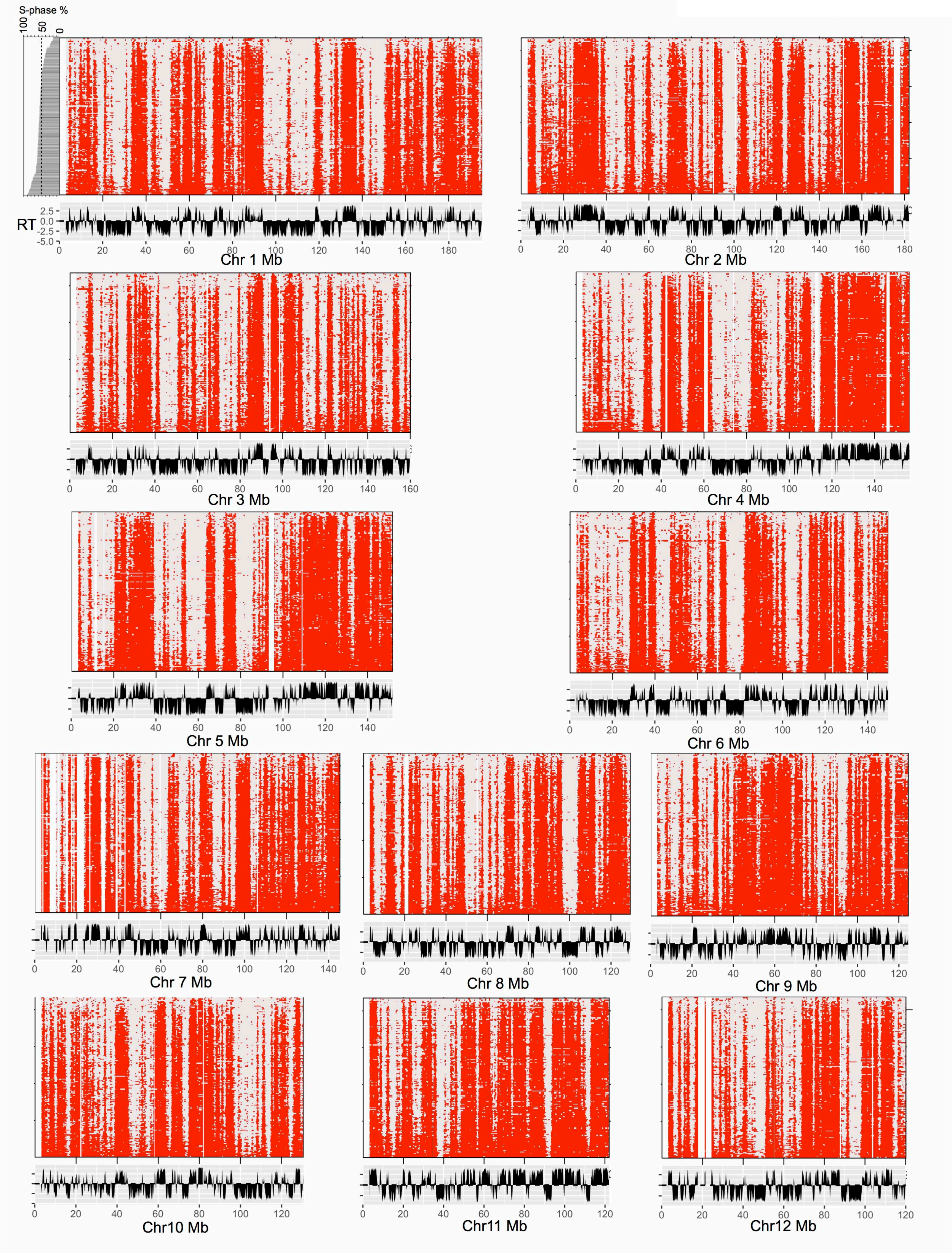
S-phase %
100
50
0
RT
2.5
0.0
-2.5
-5.0
Chr 1 Mb
Chr 2 Mb
Chr 3 Mb
Chr 4 Mb
Chr 5 Mb
Chr 6 Mb
Chr 7 Mb
Chr 8 Mb
Chr 9 Mb
Chr10 Mb
Chr11 Mb
Chr12 Mb

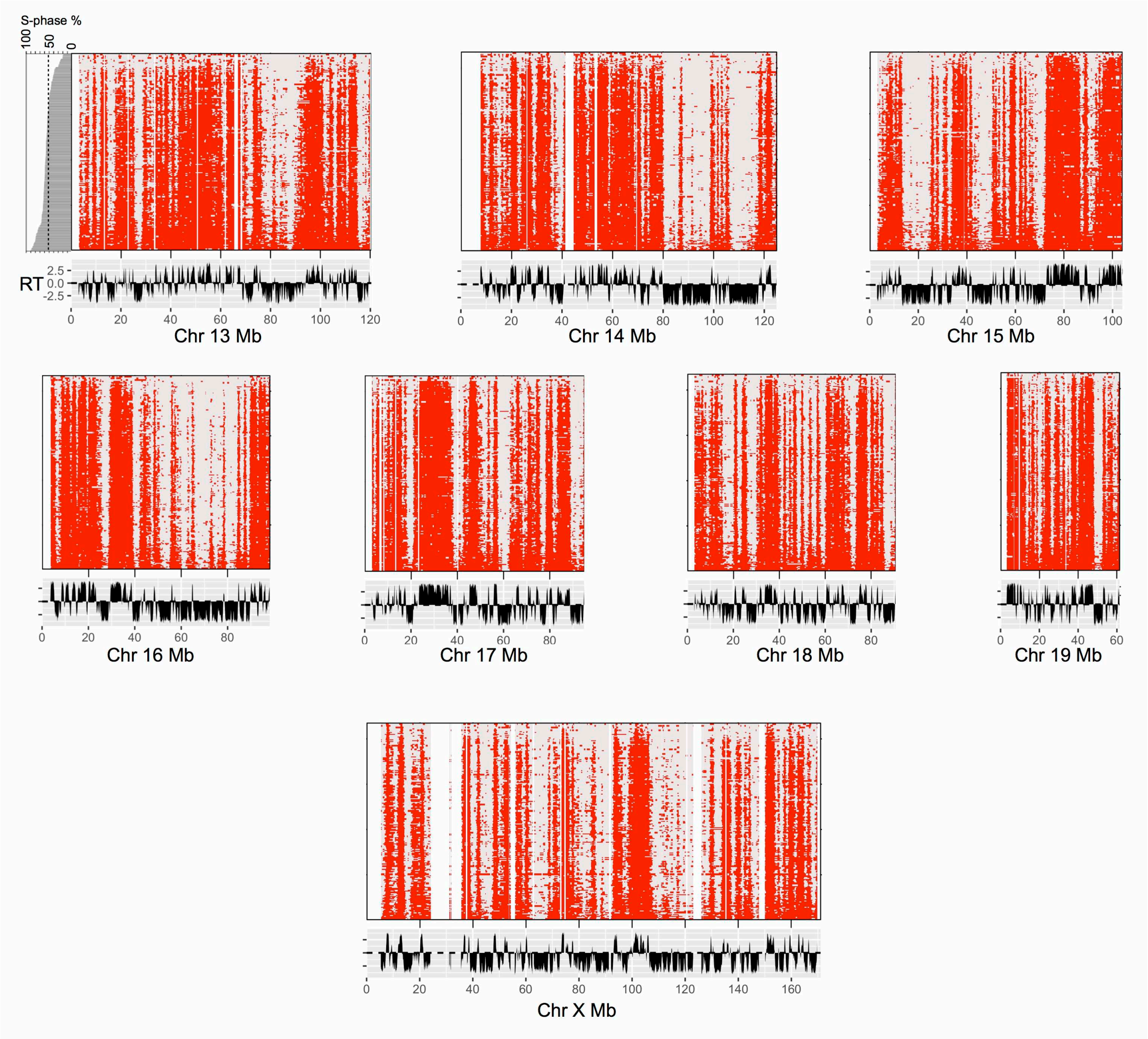

Supplementary Figure 6: Binararized data ranked by progress in S-phase for 75 haploid cells and 71 diploid cells parsed into paternal and maternal genomes for all chromosomes.

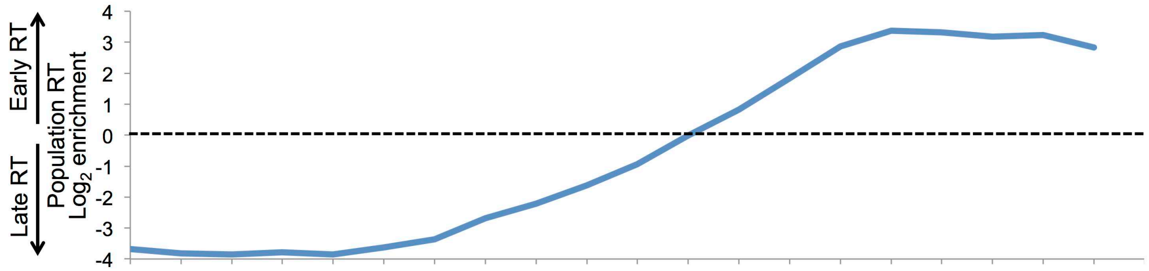

50 kb bin positions (Mb)	4	4.05	4.1	4.15	4.2	4.25	4.3	4.35	4.4	4.45	4.5	4.55	4.6	4.65	4.7	4.75	4.8	4.85	4.9	4.95
Population RT: RT log_2 scale	-3.68	-3.81	-3.86	-3.79	-3.85	-3.62	-3.36	-2.68	-2.22	-1.62	-0.94	-0.02	0.82	1.84	2.86	3.38	3.32	3.18	3.23	2.83
Population RT: RT 10 h scale	9.21	9.59	9.68	9.52	9.67	9.03	8.24	6.99	6.44	5.79	5.11	4.28	3.53	2.65	1.65	0.99	1.08	1.28	1.21	1.69

Time from scheduled replication = **Population RT 10 hour scale for each bin** - **S-phase Progression (h) for the specific single-cell**

Cell 1 (20% into S-phase, 2 Hr into S-phase)	7.21	7.59	7.68	7.52	7.67	7.03	6.24	4.99	4.44	3.79	3.11	2.28	1.53	0.65	-0.35	-1.01	-0.92	-0.72	-0.79	-0.31
Cell 2 (50% into S-phase, 5 Hr into S-phase)	4.21	4.59	4.68	4.52	4.67	4.03	3.24	1.99	1.44	0.79	0.11	-0.72	-1.47	-2.35	-3.35	-4.01	-3.92	-3.72	-3.79	-3.31
Cell 3 (80% into S-phase, 8 Hr into S-phase)	1.21	1.59	1.68	1.52	1.67	1.03	0.24	-1.01	-1.56	-2.21	-2.89	-3.72	-4.47	-5.35	-6.35	-7.01	-6.92	-6.72	-6.79	-6.31

Supplementary Figure 7: Generation of cell-specific "Time from scheduled replication". The steps involved in the generation of cell-specific "Time from scheduled replication" is shown for a 1 Mb example region. The population replication-timing values in log2 enrichment scale was converted to a 0-10 hour scale such that each 50 kb bin position has a population-based replication-timing value in hours. In this scale, the late replicating bin positions will have values close to 10 h and early replicating bin positions will have values closer to 0 h. Next for each cell, it's progression in S-phase was converted to hours. The cell-specific "time from scheduled replication" profile was generated by subtracting the number of hours the individual cell has progressed in S-phase from the population-based genome-wide replication time in hours for each 50 kb bin. Thus for each cell, positive values indicate bins that are scheduled to replicate in the future and negative values indicate bins that should have already replicated.

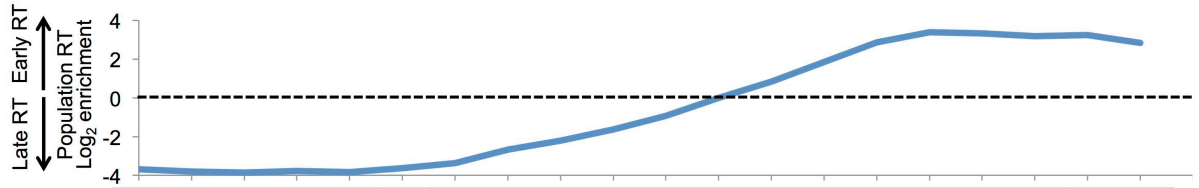

50KB bin positions (Mb)	4	4.05	4.1	4.15	4.2	4.25	4.3	4.35	4.4	4.45	4.5	4.55	4.6	4.65	4.7	4.75	4.8	4.85	4.9	4.95
Pop. RT 10 h scale	9.21	9.59	9.68	9.52	9.67	9.03	8.24	6.99	6.44	5.79	**5.11**	4.28	3.53	2.65	1.65	0.99	1.08	1.28	1.21	1.69

Mid-replicating position (~5 h)

"Time from scheduled replication"

Example cells

Cell 1																				
Cell 53 **2.48**	6.73	7.11	7.2	7.04	7.19	6.55	5.76	4.51	3.96	3.31	2.63	1.8	1.05	0.17	-0.83	-1.49	-1.4	-1.2	-1.27	-0.79
Cell 54 **3.6**	5.61	5.99	6.08	5.92	6.07	5.43	4.64	3.39	2.84	2.19	1.51	0.68	-0.07	-0.95	-1.95	-2.61	-2.52	-2.32	-2.39	-1.91
Cell 55 **5.37**	3.84	4.22	4.31	4.15	4.3	3.66	2.87	1.62	1.07	0.42	-0.26	-1.09	-1.84	-2.72	-3.72	-4.38	-4.29	-4.09	-4.16	-3.68
Cell 56 **6.42**	2.79	3.17	3.26	3.1	3.25	2.61	1.82	0.57	0.02	-0.63	-1.31	-2.14	-2.89	-3.77	-4.77	-5.43	-5.34	-5.14	-5.21	-4.73
Cell 57 **8.07**	1.14	1.52	1.61	1.45	1.6	0.96	0.17	-1.08	-1.63	-2.28	-2.96	-3.79	-4.54	-5.42	-6.42	-7.08	-6.99	-6.79	-6.86	-6.38
Cell N																				

Measured binary signal

Cell 1																				
Cell 53 **2.48**	0	0	0	0	0	0	0	0	0	0	0	1	1	1	1	1	0	0	0	0
Cell 54 **3.6**	0	0	0	0	0	0	0	0	0	0	1	1	1	1	1	1	1	1	1	1
Cell 55 **5.37**	0	0	0	0	0	0	0	0	0	0	1	1	1	1	1	1	1	1	1	1
Cell 56 **6.42**	1	1	1	1	1	1	1	1	1	1	1	1	1	1	1	1	1	1	1	1
Cell 57 **8.07**	1	1	1	1	1	1	1	1	1	1	1	1	1	1	1	1	1	1	1	1
Cell N																				

"**Time from scheduled replication**" vs. **Binary signal** for each 50 Kb position across the genome

Cell 1		
	2.63	0
	1.51	1
	-0.26	1
	-1.31	1
	-2.96	1
Cell N		

Define 0.1 h intervals for "**Time from scheduled replication**" and calculate fraction of replicated binary data points within each possible interval.

Note: Some intervals are not possible. For a mid-replicating position shown in this example (5.11 h) , possible **Time from scheduled replication are** -5 to +5 given a 10 h S-phase

Interval	Binary signal within intervals	Percentage replicated
[-10, -9.9)	Not possible	NA
[-9.9, -9.8)	Not possible	NA
⋮	⋮	⋮
[-0.1, 0)	0 0 0 1 1	40%
[0, 0.1)	0 1 0 1	50%
[0.1, 0.2)	1 0 0 1	50%
⋮	⋮	⋮
[9.8, 9.9)	Not possible	NA
[9.9, 10)	Not possible	NA

Supplementary Figure 8: Cell-to-cell extrinsic variability calculation. The goal of this analysis is to compare the cell-to-cell variability as a function of the "Time from scheduled replication". The example here shows this calculation for a mid-replicating 50 kb bin position. The "Time from scheduled replication" and the measured binary signals are collected for this bin position across all cells and the data is split into 0.1 h intervals of "Time from scheduled replication". The fraction of replicated data points is calculated for each interval. This analysis was repeated for all bin positions across the genome. Then the mean across all bin positions were plotted for each 0.1 h interval of the "Time from scheduled replication" (Fig. 3a).

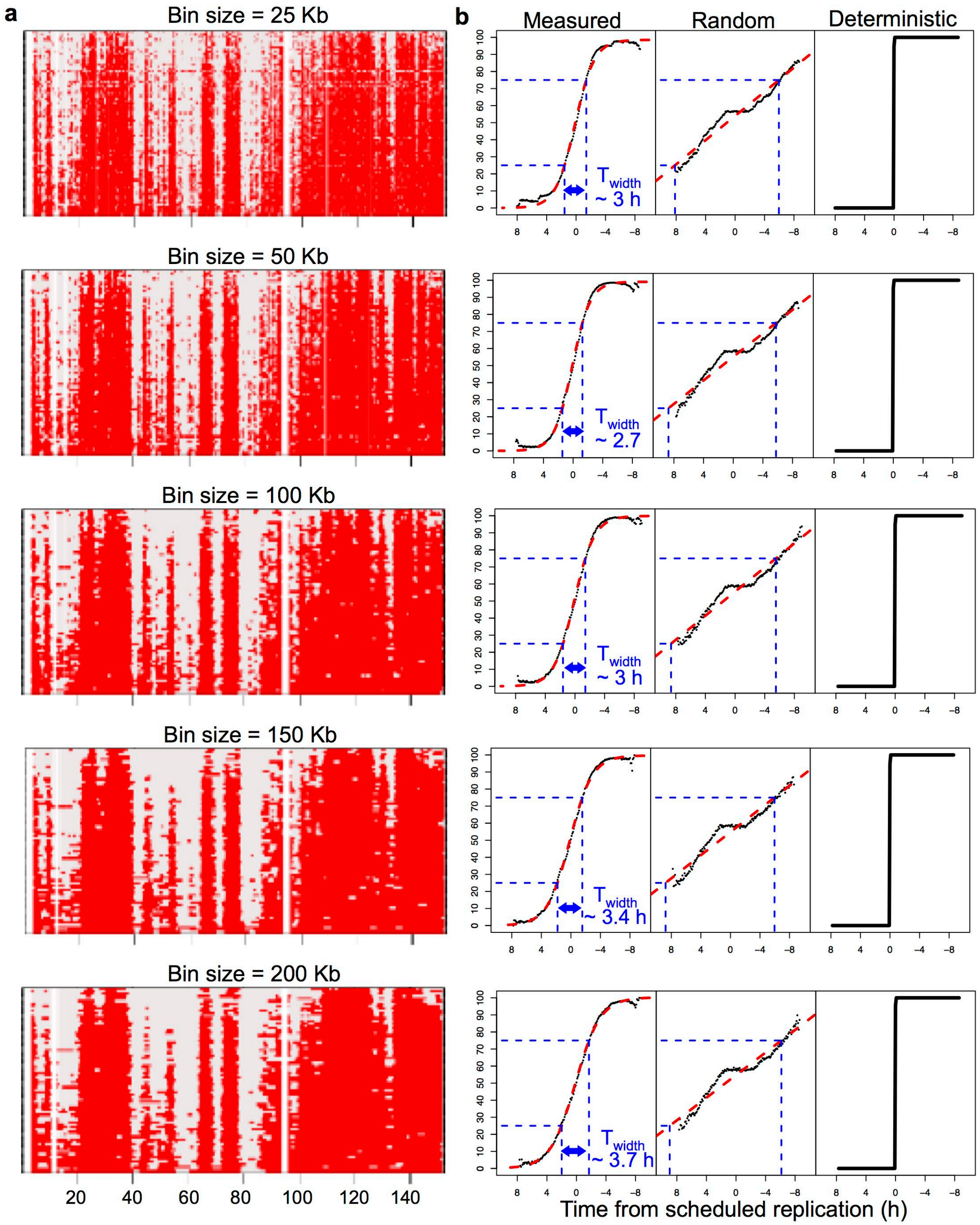

Supplementary Figure 9: Single-cell replication timing at different bin sizes. a) Binarized single-cell replication timing profiles at different bin sizes. b) Measuring single-cell replication variability similar to figure 3a at different bin sizes.
