# Supplementary material for: Single-cell replication profiling to measure stochastic variation in mammalian replication timing
Source: Nat Commun. 2018 Jan 30;9:427. doi: 10.1038/s41467-017-02800-w (PMC5789892; doi:10.1038/s41467-017-02800-w)
Supplement: Supplementary file 3 — Description of Additional Supplementary Files [file 41467_2017_2800_MOESM3_ESM.pdf]

Description of Additional Supplementary Files

File Name: Supplementary Movie 1
Description: Selecting ideal threshold for binarization of the segmented data. 100 equally spaced thresholds were used to find the ideal threshold for binarization. At each step, all bins with a segmented value above the threshold were classified as replicated and below the threshold were classified as un-replicated. Panel a shows the segmented data (red line) and binarized data (blue line) for chromosome 1. Instead of using 1 and 0 to denote the binary signal, we used the means of the replicated and un-replicated fractions (dotted vertical line, Panel b). A mixture model with 2 components was used to find the replicated and un-replicated distribution in the data. This allowed calculation of the manhattan distance between the segmented and binarized data at each step (Panel c). The threshold value at which the distance between the segmented and binarized data was minimum was chosen as the ideal threshold. The solid moving black represents the current threshold in all panels.

File Name: Supplementary Data 1
Description: Barcoded oligos from Baslan. et al, 2015 used for barconding single cells. A * in the oligo seqeunce indicates a 3'-phosphorothioate bond and a /5Phos / indicates a 5'- phosphate group.
